# Supplementary material for: Large‐scale PVA modeling of insects in cultivated grasslands: The role of dispersal in mitigating the effects of management schedules under climate change
Source: Ecol Evol. 2022 Jul 11;12(7):e9063. doi: 10.1002/ece3.9063 (PMC9272070; doi:10.1002/ece3.9063)
Supplement: Supplementary file 5 — Appendix S5 [file ECE3-12-e9063-s001.pdf]

Supplement S5: Illustration of correlation between evaluation parameters

Supplement to

> Leins J. A., Grimm V., Drechsler M. *Large scale PVA modelling of insects in cultivated grasslands: the role of dispersal in mitigating the effects of management schedules under climate change*

The scatter plots below correlate three evaluation parameters (rows) from simulations with dispersal (population size [Σ eggs], population density [eggs m^{-2}], maximum established distance [meters]) with population density [eggs m^{-2}] stemming from simulations without dispersal as well as regional grassland cover [in %] in a radius ≤ 1,500 m of a simulation run's respective habitat of origin (columns). Each colored dot represents the correlated values of INDEPENDENT simulation runs, or rather their mean over 10 replicate runs (5 without dispersal), resulting from the population dynamics and dispersal process of a SINGLE initial population in the center of one out of 107 cells. The dot colors distinguish the three climate change scenarios (CCS) Clim-FF (RCP 2.6, green), Clim-MOD (RCP 4.5, brown) and Clim-BAU (RCP 8.5, pink). The colored numbers give the correlation coefficient ρ for the respective parameter combinations by CCS. The black lines represent the respective linear regression, where dashed=Clim-FF, dotted=Clim-MOD and dash dotted=Clim-BAU.

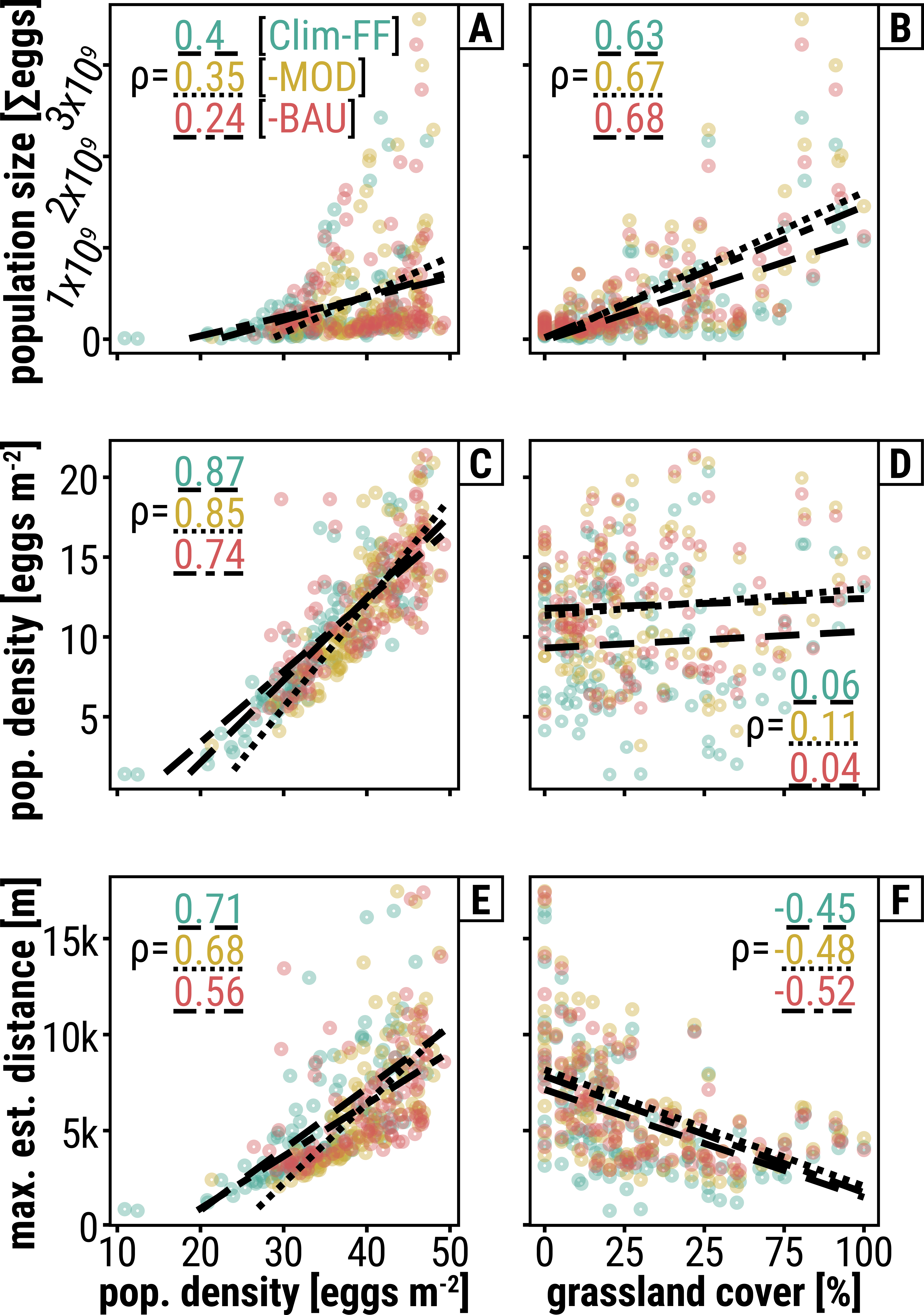

A
B
C
D
E
F
ρ= 0.4 [Clim-FF]
0.35 [-MOD]
0.24 [-BAU]
ρ= 0.63
0.67
0.68
ρ= 0.87
0.85
0.74
ρ= 0.06
0.11
0.04
ρ= 0.71
0.68
0.56
ρ= -0.45
-0.48
-0.52
population size [Σeggs]
0
1x10⁹
2x10⁹
3x10⁹
pop. density [eggs m⁻²]
5
10
15
20
max. est. distance [m]
0
5k
10k
15k
10
20
30
40
50
pop. density [eggs m⁻²]
0
25
25
75
100
grassland cover [%]
